# The Influence of Polylactic Acid Filament Moisture Content on Dust Emissions in 3D Printing Process

**DOI:** 10.3390/s24247890

**Published:** 2024-12-10

**Authors:** Anna Karwasz, Filip Osiński, Weronika Kaczmarek, Kacper Furmaniak, Izabela Rojek

**Affiliations:** 1 Faculty of Mechanical Engineering, Poznan University of Technology, Piotrowo 3, 60-138 Poznań, Poland; anna.karwasz@put.poznan.pl (A.K.); filip.osinski@put.poznan.pl (F.O.);; 2 Faculty of Computer Science, Kazimierz Wielki University, Chodkiewicza 30, 85-064 Bydgoszcz, Poland

**Keywords:** additive manufacturing, filament moisture, PLA, dust emissions, laser dust sensors

## Abstract

This paper presents the results of a study on the effect of moisture content in polylactic acid (PLA) filaments on dust emissions during incremental manufacturing. The tests were conducted in a customised chamber using a standard 3D printer, and Plantower PMS3003 sensors were used to monitor air quality by measuring PM1, PM2.5 and PM10 concentrations. The filament humidity levels tested were 0.18%, 0.61% and 0.83%. The results show that a higher moisture content in the filament significantly increases dust emissions. For dry filaments (0.18% humidity), the average dust emissions ranged from 159 to 378 µg/m^3^. Slightly humid filaments (0.61%) produced higher emissions, with averages between 59 and 905 µg/m^3^, with one outlier reaching up to 1610 µg/m^3^. For very humid filaments (0.83%), the highest average emissions were observed, ranging from 57 to 325 µg/m^3^, along with greater variability (standard deviation up to 198). These findings highlight that increased filament humidity correlates with elevated dust emissions and greater instability in emission levels, raising potential health concerns during 3D printing.

## 1. Introduction

Three-dimensional printing is gaining more and more in terms of applications as time goes by. Today, products made using this technology can be found in aerospace, medicine and even everyday objects. According to the 3D Printing Trend Report 2023 [1] conducted by Hubs (Protolabs Network), 66% of the products created via 3D printing are intended for prototyping. According to the same report, the FDM method is the most preferred method for 3D printer users at over 55%.

The increase in interest in FDM incremental manufacturing technology in recent years is due to its numerous advantages. These include the possibility of using various materials such as polypropylene, ABS, PLA, HIPS, PTU and PA. Another important feature is the creation of complex shapes or the possibility of integrating the design and production phases into a single process, which translates into a reduction in the time needed for the construction and development of a product [2]. In the case of the FDM method, it is also necessary to recognise the disadvantages of this process, such as sensitivity to changes in the process parameters or dust emissions affecting the operator and the environment [3].

The use of incremental manufacturing technology has been noted in the chemical and pharmaceutical industries, among others [4,5]. In medicine, there is the printing of models used to prepare and practice before operations and procedures, and the production of diagnostic tools [6]. In the mechanical industry, its use in the manufacture of batteries as energy storage is extremely important with the increasing dominance of sustainable energy sources [7]. It takes approximately four–five weeks to manufacture turbine blades for an engine using a traditional method (e.g., making a casting). Using 3D printing, this process can be shortened to as little as one day [8].

An important issue for the material is the selection of an appropriate head temperature [9]. If the temperature is very low, the material does not liquefy, the coefficient of viscosity and the torque of the filament feeder motor are increased, and in extreme situations, the head even becomes clogged. If the temperature is too high, the material particles degrade, and the filament becomes rough.

A number of factors influence the quality of the print and the progress of the FDM incremental manufacturing process, such as the thickness of the layer; the temperature of the head; and the temperature of the extruded material, its moisture content or the orientation of the print [10]. The properties of the filaments—which are important in terms of the quality of FDM products—are determined by the glass transition temperature, material shrinkage, thermal resistance, mechanical strength, elasticity, solubility, food contact approval and the emission of harmful vapours, among other things [11]. It was the harmfulness of vapours emitted during 3D printing that prompted the authors of this study to look more closely at this issue.

Particle excitement is influenced by the temperature of the print head. The higher the print temperature, the more particles are excited [12,13,14]. In the case of PLA, increasing the temperature to 220 °C resulted in a significant increase in particle excitement, which was caused by material degradation.

Ding et al. [15] described the mechanism of emission formation in filaments made of PLA, ABS and PVA. They used thermogravimetric analysis (TGA) to simulate the heating process of the material in the head and the evolved gas analysis (EGA) approach. It was observed that emission starts during glass transition and peaks at the melting point of the material. Each of the filaments started emitting VOCs at temperatures ranging from 75 °C to 150 °C, which were temperatures higher than the glass transition temperature and lower than the temperatures used during 3D printing.

According to the printer manufacturer Prusa, damp filaments can cause poor print quality due to poor layer adhesion and the formation of thickening or evaporation during material extrusion. Most materials used in 3D printing are hygroscopic, so they should be adequately protected from moisture, as drying the filament should be a last resort [16]. This was confirmed in the study by Hamrol et al. [17,18], which noted that the moisture content of ABS filaments has a negative impact on the quality of the products produced. The authors [19] noted that as the layer thickness increases, particle emissions increase, but increasing the layer density has the opposite effect, reducing particle concentration by 33%. By selecting the appropriate parameters mentioned in this paragraph, according to the researchers, it is possible to reduce emission values by 96%. Azimi et al. [20] investigated emissions for commercial 3D printers using filaments of various materials, including PLA, which had the lowest emission rate but emitted lactide, which can cause eye and skin irritation. The paper’s authors also compared the effects of print orientation and shape (requiring similar print times) on particle counts, and no significant differences were seen.

The use of a 3D printing method such as FDM technology is not detached from the environment. Attention has also been drawn to the impact of incremental manufacturing on the printer operator or his working environment.

Steinle [21] measured and characterised particulate emissions during FDM printing in poorly and well-ventilated rooms using PLA and ABS filaments. In the poorly ventilated room, particulate and VOC emissions were higher for both materials than in the well-ventilated room. The emissions consisted mainly of VOCs, but soot-like particles that formed agglomerates were also noted. In the case of PLA, methyl methacrylate (MMA) was detected, which was even present in the poorly ventilated room long after the printing process was completed. MMA is a respiratory and skin irritant. Zinc and iron particles were also noticed in the air, which may have been caused by the abrasion of parts in the printer.

VOC particles emitted during 3D printing can irritate throat mucous membranes and cause cardiovascular disease and even strokes. Karwasz et al. [11] present a simulation showing the spread of contaminants when the 3D printer room door is open and in the room just after production. Currently, no legislation regulates the conditions under which the printers should be used. Particles are released when the filament is heated in the printer head. VOCs evaporate more or less quickly at room temperature. The WHO has divided them into three categories based on their boiling points: very volatile organic compounds (VVOCs), volatile organic compounds (VOCs) and semi-volatile organic compounds (SVOCs).

Most of the dust emitted during the FDM process forms agglomerates resembling spherical particles on the nanoscale. An increased printing temperature results in an increase in the number of particles emitted. For VOC particles, several correlations have been noted. Different filaments cause different emissions, and some of them reach or exceed the permitted level of possible occupational exposure. Depending on the analytical method used, different results have been obtained for the same materials, demonstrating the need to standardise test methods. The authors point out that studies on dust emissions are currently under development, as only some printer models and types of filament have been tested. They also conducted an in vitro experiment on human airway epithelial cells, which resulted in increased cytotoxicity, oxidative stress, inflammation and even necrosis [22].

Many researchers have conducted studies to find a correlation between 3D printing and users’ health problems [23,24]. Forty-six people using FDM printing technology in 17 different companies were involved in experiments. A total of 59% of respondents recorded respiratory complaints occurring at least once a week throughout the year. Employees using the devices 40 h a week were more likely to be diagnosed with allergic rhinitis or asthma. More than half (52%) of users reported not using any personal protective equipment. Other authors [25] looked at the story of a patient who developed asthma after starting to work with 3D printers. He had no health problems prior to starting work, only suffering from asthma-like symptoms as a child. The operator used ABS filament prints, and the workspace had a volume of 3000 cubic feet and accommodated 10 devices. After 10 days on the job, the patient reported coughing, shortness of breath and chest tightness. The other five co-workers did not report such symptoms.

Kim et al. [26] investigated the amount of formaldehyde released and PM10 and PM2.5 concentrations during 3D printing on a printer located in an office. The workspace consisted of 3D printers, two ceiling fans, air conditioning, wall fans and a measuring device. The room, measuring 5.0 m × 3.0 m × 2.5 m, was maintained at temperatures of 18–22 °C. The second measuring sensor was located in an office area located behind a closed workspace door, equipped with a window and an environment free of cigarette smoke, perfume and food. Films made of PLA, ABS, TPU, Dental LT Clear and Flexible 80A were tested. In Table 1, the results obtained for PLA, among others, are summarised.

The results of the experiment show that the standards set by the WHO were exceeded. The effect of ventilation was shown to be positive, and there was a decrease in the concentration of harmful compounds when the ventilation was switched on [26].

The smaller the printing room or the more printers in the room, the higher the concentration and the amount of formaldehyde released and the exceedance of PM10 and PM2.5 [27,28,29,30,31]. Measurements of VOCs in all studies were higher than the standards set by the National Institute for Occupational Safety and Health (NIOSH).

Emissions during the 3D printing process are not limited to particulate matter and volatile organic compounds (VOCs); odorous emissions also play a significant role in affecting the working environment and operator comfort [32]. The concentration of odours and the concentration of volatile organic compounds can be given in ppb, with the average, for example, N-butanol odour threshold being in the range of 20–80 ppb (40 ppb represents the accepted odour threshold for n-butanol), and the standard deviation of individual responses is less than 2.3. Materials like PLA are often described as emitting a “sweet” smell during printing due to the thermal degradation of the polymer and the release of specific chemical compounds [11,33].

Steinle [21] observed that during printing with PLA, methyl methacrylate (MMA) was detected, which is known for its distinctive odour and potential to cause respiratory and skin irritation. Odorous emissions can persist in the workspace long after the printing process has concluded, especially in poorly ventilated areas.

Increased filament moisture may influence the intensity of odorous emissions, as the presence of water in the material can lead to additional degradation pathways and the formation of new reaction products with strong odours [34]. Studies highlight that proper ventilation and control of process parameters are crucial not only for reducing particulate and VOC emissions but also for minimising unpleasant odours [26].

Considering odorous emissions is essential when evaluating the overall impact of the 3D printing process on the working environment and operator health. Therefore, investigating the effect of filament moisture content on emissions during PLA printing is important not only from the perspective of particulate matter but also regarding the potential odorous compounds that may accompany the process [35].

Recent studies have highlighted emissions from additive manufacturing, focusing on different filament types and process parameters. For instance, PLA filaments generate lower levels of volatile organic compounds (VOCs) compared to ABS but still pose health risks due to the release of irritants like lactide. Emissions tend to increase significantly when filaments are heated beyond their glass transition temperature.

While research has explored factors like ventilation [21], limited attention has been paid to the impact of filament humidity. Studies on ABS suggest that moisture negatively affects print quality and increases emissions [17,18]. However, the role of PLA filament moisture in particle size-specific emissions (PM1, PM2.5 and PM10) remains insufficiently addressed.

This study investigates how varying levels of PLA filament humidity influence dust emissions under different printing temperatures. By focusing on specific particle size fractions, it provides new insights into emission variability and its implications for operator safety and environmental impact. These findings aim to guide safer and more sustainable practices in additive manufacturing.

## 2. Materials and Methods

### 2.1. Filament Characteristics

A PLA filament with the trade name Spectrum Premium PLA was used in the study. It is characterised by resistance to material warping, biodegradability, low odour and a relatively low price. In addition, it has very good mechanical strength and exhibits higher impact strength. Table 2 summarises the properties of the Spectrum Premium PLA filament [36].

Our research started with the proper preparation of the filament, in the case of dry material, by soaking it. The FDM 3D printing process was then performed, during which dust emissions were recorded. Printing took place at three different head temperatures, 185 °C, 200 °C and 215 °C, respectively.

The Spectrum Premium PLA filament was subjected to a process of soaking in distilled water in a sealed container. Depending on the desired effects, the soaking time was varied. The moisture content of the material was measured using a weighing machine. Table 3 shows information on the filaments used.

### 2.2. 3D Printing Properties

**Figure 1:**
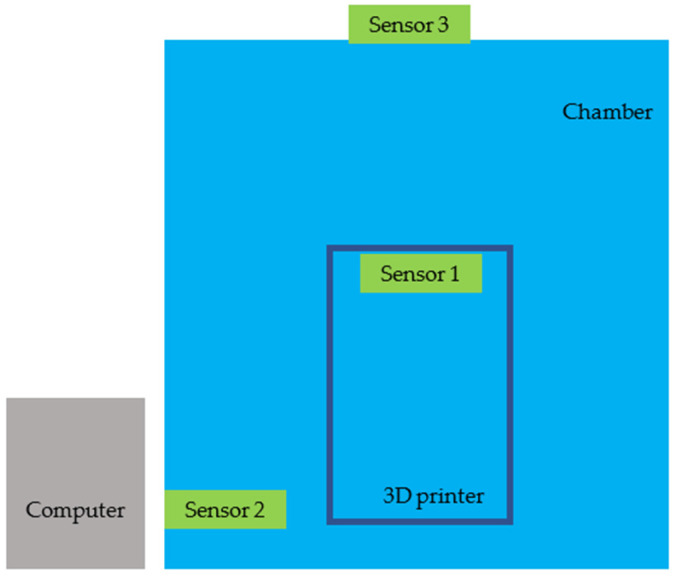
Schematic of the test bench.

The printing process was carried out using an Anycubic S printer placed in a specially prepared chamber (Table 4) [38]. Particles were measured using two sensors inside the printing chamber and one outside the chamber, as shown in Figure 1.

### 2.3. Research Plan

The printing process took place using three variants due to the different printing temperatures of 185 °C, 200 °C and 215 °C. In each case, the working table temperature was 60 °C, and the filament feed rate was 50 mm/s. On average, the printing process lasted about 80 min, and during the process, the amount and size distribution of the dust released by the PLA was recorded, as described in more detail in the next subsection. Given the difference in head temperatures and the soaking time of the filament, the sample determinations are summarised in Table 5.

In the incremental manufacturing process, a three-dimensional product is obtained. For this research, the decision was made to produce a research paddle in accordance with ISO 527-1A [40]. Its advantages include a low-complexity design and a relatively short printing time. Printed objects are used for tensile strength testing of materials, in which they are destroyed, so an uncomplicated shape is highly desirable.

### 2.4. Testing Setup

Various sensors have been employed in previous research to monitor particulate emissions during additive manufacturing processes. Common types include optical sensors, gravimetric sensors and spectroscopic analysers [41,42,43,44,45]. Optical sensors, such as laser-based devices, are widely used due to their ability to provide real-time measurements of particulate matter (PM) concentrations in different size ranges (PM1, PM2.5 and PM10) [44,45]. Gravimetric methods, though highly accurate, are labour-intensive and do not offer real-time results [41,45]. Spectroscopic analysers, while highly sensitive, are typically more expensive and less practical for small-scale setups [42,43].

Gravimetric methods are often considered the gold standard for PM measurement due to their high accuracy, as they rely on collecting particles on filters and weighing them to determine concentration. However, these methods are labour-intensive, do not allow real-time monitoring and are not practical for dynamic processes like 3D printing. Optical sensors, such as the PMS3003, offer a practical alternative by providing continuous measurements, even though they are less precise for ultrafine particles compared to gravimetric techniques.

The PMS3003 sensor was chosen for this study due to its specific advantages in monitoring particulate emissions. It is a laser-based optical sensor capable of detecting particles across multiple size ranges (PM1, PM2.5 and PM10) with high sensitivity. This technology allows for the rapid detection of airborne particles and provides real-time data, which is crucial for capturing dynamic changes during the printing process [41]. The PMS3003’s compact design, ease of integration with data acquisition systems and relatively low cost make it particularly suitable for research in small-scale environments, such as the customised 3D printing chamber used in this study.

Another advantage of the PMS3003 is its broad operating range, with a working temperature range of −5 °C to 60 °C, ensuring consistent performance, even in varying environmental conditions. Its ability to detect particle concentrations as low as 0.3 µm and its short response time (under 10 s) provide the resolution and speed necessary for accurately monitoring emissions during incremental manufacturing processes [46].

While the PMS3003 has notable advantages, it is not without limitations. Like other optical sensors, its performance can be affected by factors such as high humidity or particle aggregation, which may impact measurement accuracy [47]. However, these potential drawbacks were mitigated in this study by maintaining controlled environmental conditions inside the chamber and using additional sensors (e.g., DHT22) to monitor and adjust for humidity.

The decision to use the PMS3003 instead of other available sensors was primarily driven by the need for a balance between accuracy, cost and practicality [38]. While more advanced spectroscopic or gravimetric methods could theoretically offer higher precision, their complexity and cost would exceed the requirements of this study. The PMS3003 provided reliable and reproducible measurements that align with the objectives of investigating the relationship between PLA filament moisture and particulate emissions.

By utilising the PMS3003, this research demonstrates the feasibility of using cost-effective and real-time monitoring tools for assessing particulate emissions in additive manufacturing, particularly in environments where gravimetric methods would be impractical.

The test rig consists of sensor 1 and sensor 2, located together with the printer in the chamber, so they were responsible for measuring particles directly from the printer. Sensor 3 tested the concentration of particles in the environment as it was located outside the chamber. All three sensors were connected to a computer, which stored the results in a text file.

The study used Plantower’s PMS3003 sensors to monitor air conditions and measure PM1, PM2.5 and PM10. Their measurement relies on an optical method to study the composition of particulate matter.

A laser beam falls on the pollutants appearing in the measurement chamber and is scattered, and a photodetector detects the particles. An internal processor analyses the signal coming from the photosensitive element. Table 6 shows the basic data for this device [46].

The digital data that the sensor sends to the host device are readings of the concentration of airborne particles in three ranges, PM1.0, PM2.5 and PM10.0 and the unit µg/m^3^. In the case of large instantaneous fluctuations, measurements are sent every 800 ms, while for small fluctuations, the measurement result is sent every 2.3 s to obtain more reliable data [47].

**Figure 2:**
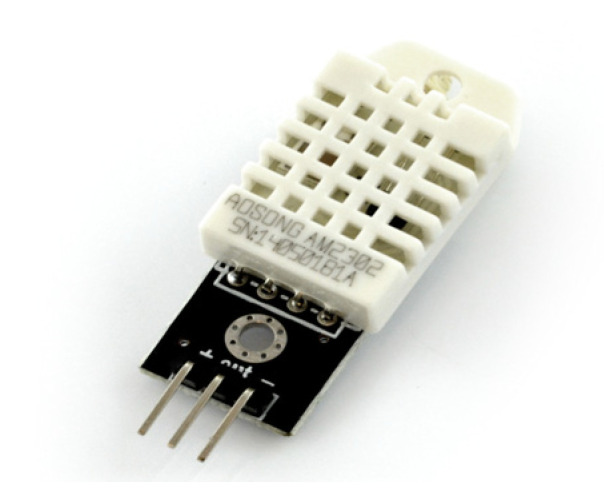
Measuring module with DHT22 sensor [48].

When performing particle concentration measurements on the constructed bench, information on the temperature and humidity prevailing inside is important. For this purpose, a proven module containing a DHT22 sensor was selected, as shown in Figure 2.

The module is characterised by its compact dimensions, has a large operating range (−40 °C to +80 °C and 0–100% RH) and, importantly from the perspective of bench design, does not require additional external components. To use it, only three wires need to be connected: power, ground and the digital data output to the microcontroller. The DHT22 sensor is capable of transmitting a signal up to 20 m, which allows it to be freely placed inside the chamber. Temperature and humidity readings are sent to the master, on average, every 2 s. However, the measurement accuracy is ±0.5 °C for temperature and ±2% RH for humidity. All the described characteristics of the DHT22 sensor module allowed it to be used in the designed stand [49].

During the selection of the control unit, one of the development boards of the Arduino platform was used. This platform was chosen due to the affordability of the development environment [50], which allows the writing of any program using numerous libraries created by other users. Most models in the Arduino family use AVR microcontrollers. It was decided to use the Arduino MEGA 1280 board, on which the ATmega 1280 microcontroller was placed. This is one of the most extensive Arduino models.

**Figure 3:**
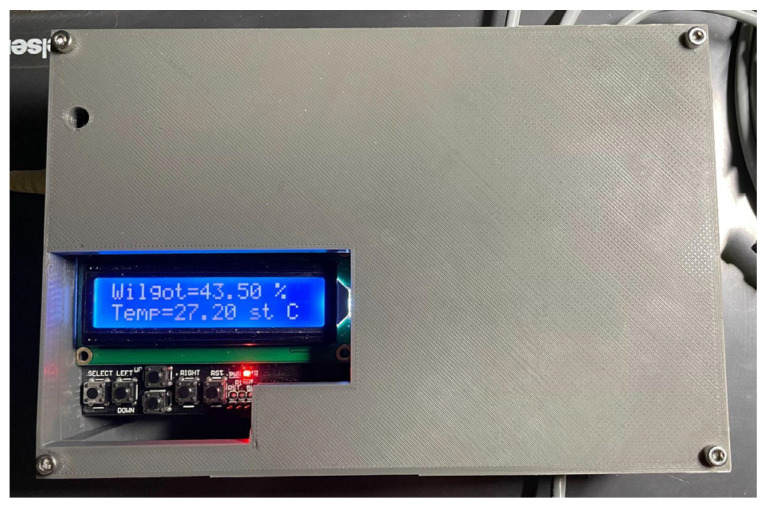
Measuring device.

All the components were put together and placed in a compact box as the next step, as shown in Figure 3.

The lid of the device was designed to allow the best possible access to the device’s control buttons. The next step was to prepare the entire device control software (Arduino Mega 1280 Library for Proteus V2.0). In the final software version (Arduino Mega 1280 Library for Proteus V2.0), a device control application was prepared.

The first part of the program contains the libraries used in it. Each of the libraries contains instructions prepared by other users to achieve the desired goal in a faster and simpler way, e.g., to read the current temperature indication from the DHT22 sensor.

In the design of the measurement device, the libraries used are LiquidCrystal [51], which allows text to be displayed on the screen of the Arduino overlay; PMS [52], which allows the efficient operation of sensors that detect particles in the air, and DHT [53], which allows the current temperature and humidity to be read from the sensor.

The next part that can be separated in the program is the setup function. This function allows an action to be performed each time the power to the device is switched on only once. It starts all four UART interfaces and initialises the temperature/humidity sensor and the screen. The sample time variable corresponds to the number of seconds it takes for a measurement from the sensors to be sent to the computer. It only accepts positive integer values. Changing it to a value of 2 results in measurements being sent to the computer at intervals of at least two seconds.

Once the device is fully initialised, the program proceeds to execute the main loop. When writing the program, care was taken to ensure that the main loop could be divided into three basic members. The first member was responsible for reading inputs to the Arduino, such as temperature and humidity. The second part of the main loop of the program was dedicated to programming the up and down buttons below the screen. Pressing the up button decreases the menu variable by one. The third part of the code was devoted to handling the display. The content of the screen was refreshed every second, but what was displayed on the screen depended on the value of the menu variable. This variable was increased or decreased depending on the buttons pressed below the screen. This solution was chosen because the screen was too small and could not hold all the information from the sensors in one composition.

The last part of the main loop was prepared to send the data going to the Arduino to the computer connected to the measuring device via a USB cable. The data sending was performed according to the setting of the sample time variable. All data were separated by a semicolon. This solution allowed the collected information to be easily imported into any data analysis program, such as Microsoft Excel 2016.

As a reminder, PM1 is particulate matter with a diameter of less than 1 µm. PM2.5 is particulate matter with a diameter of 2.5 µm or less, and it is this type of dust that, according to the WHO, is the most harmful to humans. It is formed either directly or through the aggregation of smaller particles or as secondary particles during a chemical reaction. PM2.5 is responsible for the deaths of 60,000 people a year [54]. PM10 is particulate matter with a diameter of less than 10 µm, which is also a component of smog and has adverse effects on the human respiratory system [55].

## 3. Results and Discussion

### 3.1. Overview of Results

Three trials were carried out for each experiment to obtain reproducible results. Exponential curves of the dependence of dust concentration on the duration of the 3D printing process were plotted for sensor 1 and sensor 2 and each particle size (PM1, PM2.5 and PM10). The next step was to determine the difference in concentration between the starting point and point 4600 (the end of printing). In addition, the individual results were collated into a single sheet for data comparison. Table 7 shows the results of the tests.

For experiment 5, the third trial was discarded due to erroneous results. The differences in individual trials within a single experiment may be due to testing on different days when the humidity and air temperature were different. In addition, the significant standard deviation values, which are higher for the wet filament, may be confirmation of the uneven absorption of water by the filament.

### 3.2. Temperature-Dependent Analysis of Emissions

**Figure 4:**
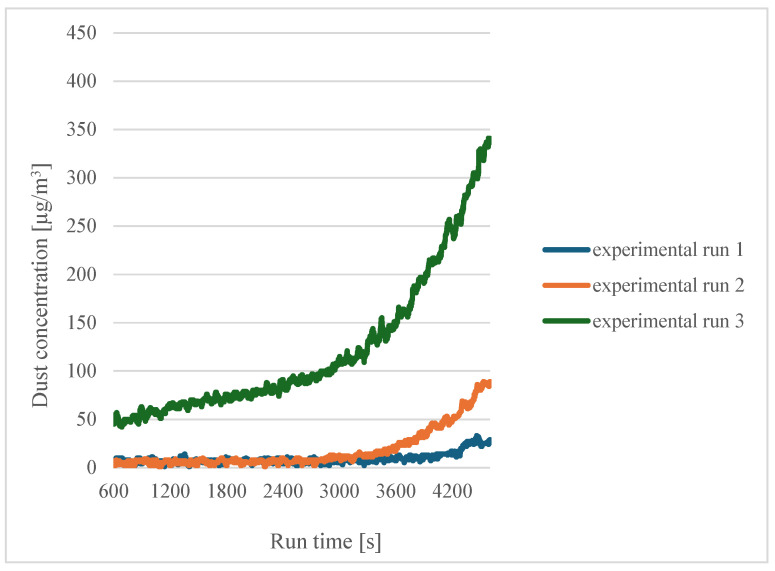
Dependence of dust concentration increase on process duration for sensor 1 for dry filaments.

In the case of dry filaments (experiments 1, 2 and 3), an increase in dust emissions can be seen as the printing temperature increases. Figure 4 shows a summary of the curves for sensor 1 in the case of dry filaments.

In Figure 4, a significant increase in concentration can be seen for experiment 3 (a printing temperature of 215 °C). For experiment 2 (a printing temperature of 200 °C), an increase in concentration is also noted compared to experiment 1 (a printing temperature of 185 °C), but it is smaller. From this, it can be concluded that an increase in printing temperature causes an increase in dust concentration and that approaching the PLA degradation temperature of 230 °C causes a sharp increase in dust concentration. A similar conclusion was reached by Karayannis et al. [12] and Deng et al. [13].

**Figure 5:**
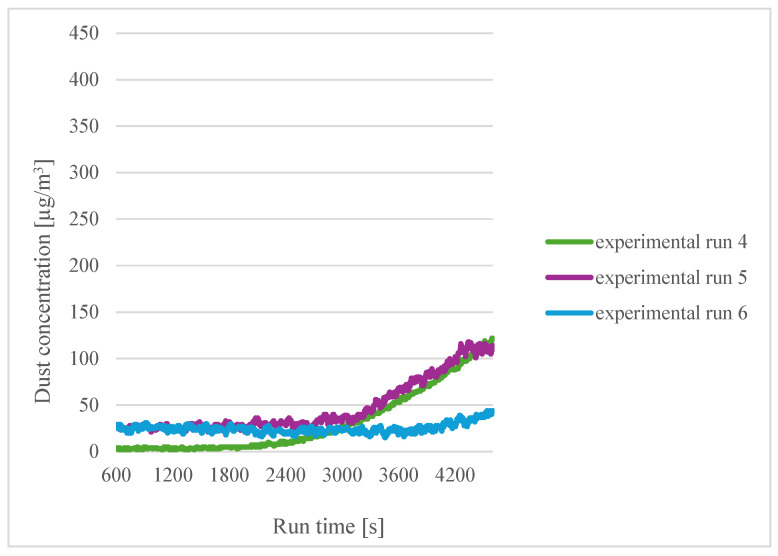
Dependence of dust concentration increase on process duration for sensor 1 when the filaments were soaked for three hours.

A similar comparison was made for the filaments soaked in distilled water for three hours (Figure 5).

At the beginning of the process for the lower printing temperature (experiment 4), the particle concentration was lowest, but as the process continued, it reached the highest value of all the experiments for a given filament moisture content. This may be related to the formation of a layer on the surface of the filament by the water, and in the case of the lower temperature, it took longer for the water to evaporate; thus, the particle concentration increased more rapidly. Interestingly, the wetted filament, in the case of the highest temperature, obtained the lowest concentration, which would indicate a beneficial effect of soaking the filament.

**Figure 6:**
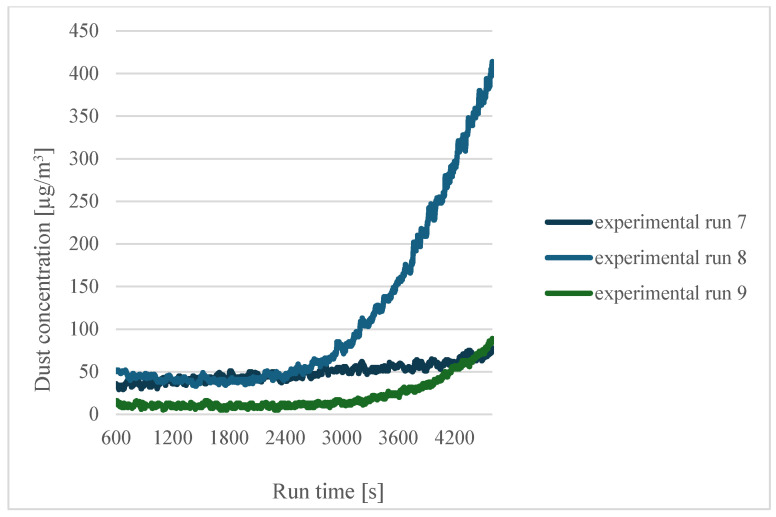
Dependence of dust concentration increase on process duration for sensor 1 when the filaments were soaked for twelve hours.

A further analysis was carried out for the filaments soaked in distilled water for twelve hours. Figure 6 shows the curves for sensor 1.

### 3.3. Moisture-Dependent Analysis of Emissions

In the case of filaments soaked for twelve hours, the highest concentration values were obtained for a printing temperature of 200 °C (experiment 8). However, for the highest printing temperature, the concentration values were significantly lower, which shows the positive effect of printing using a moist filament.

**Figure 7:**
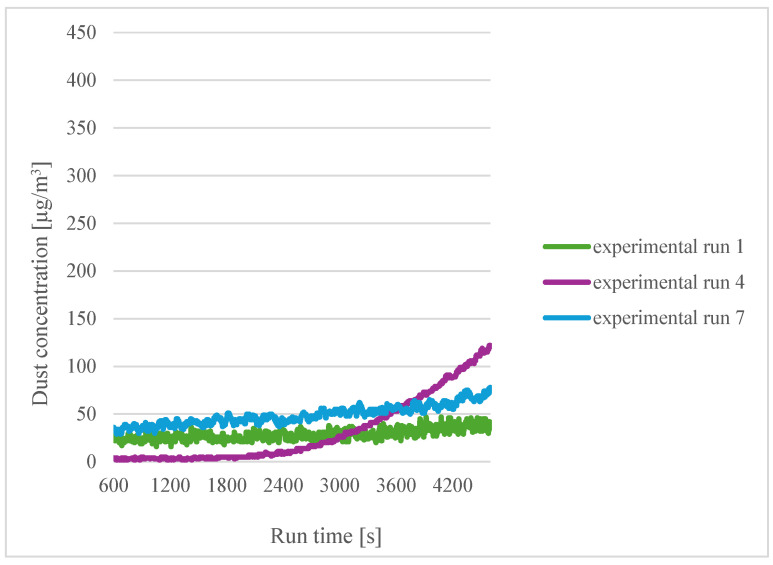
Dependence of dust concentration increase on process duration for sensor 1 for a printing temperature of 185 °C.

Comparisons were then made for different printing temperatures and different filament moisture contents. The results for a printing temperature of 185 °C for sensor 1 were collated first (Figure 7).

For a printing temperature of 185 °C, the highest emissions were obtained for the filament soaked for three hours in distilled water (experiment 4). For the other filament moisture contents, similar results were obtained. From this, it can be seen that the moisture content of the material has no effect on the emissions.

**Figure 8:**
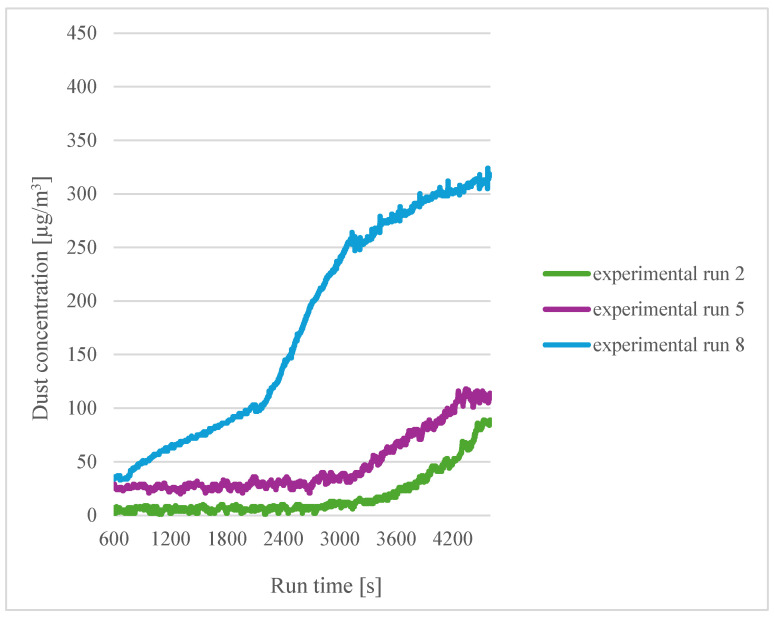
Dependence of dust concentration increase on process duration for sensor 1 for a printing temperature of 200 °C.

A similar comparison was made for a printing temperature of 200 °C (Figure 8). In this experiment, the highest emissions were obtained for the filament soaked in distilled water for twelve hours (experiment 8). For experiments 2 and 5, similar results were obtained. As for the temperature of 185 °C, there is no correlation between the moisture content of the material and the amount of emission.

**Figure 9:**
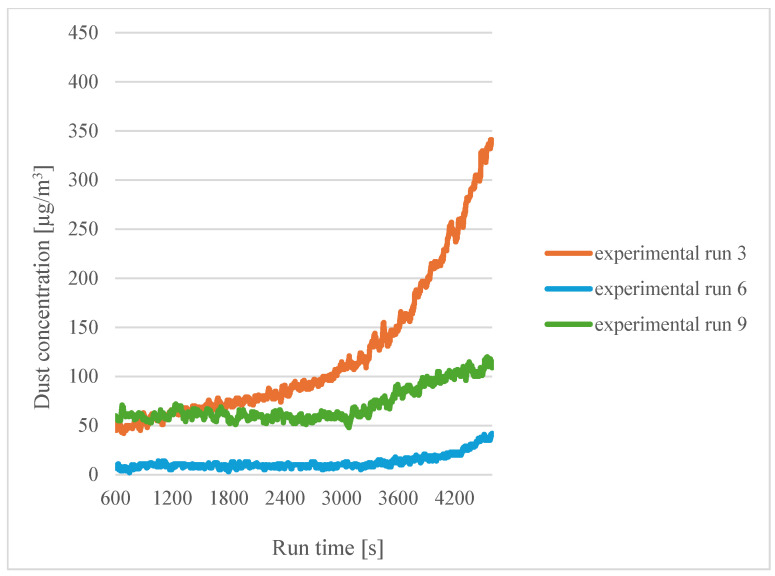
Dependence of dust concentration increase on process duration for sensor 1 for a printing temperature of 215 °C.

For a printing temperature of 215 °C (Figure 9), the highest emissions were obtained for the dry filament (experiment 3). On this basis, it can be concluded that, for the temperature limit for PLA processing, the use of wet filament is advantageous in terms of lower dust emissions.

### 3.4. Effect of Humidity on Emitted Particle Fractions

**Figure 10:**
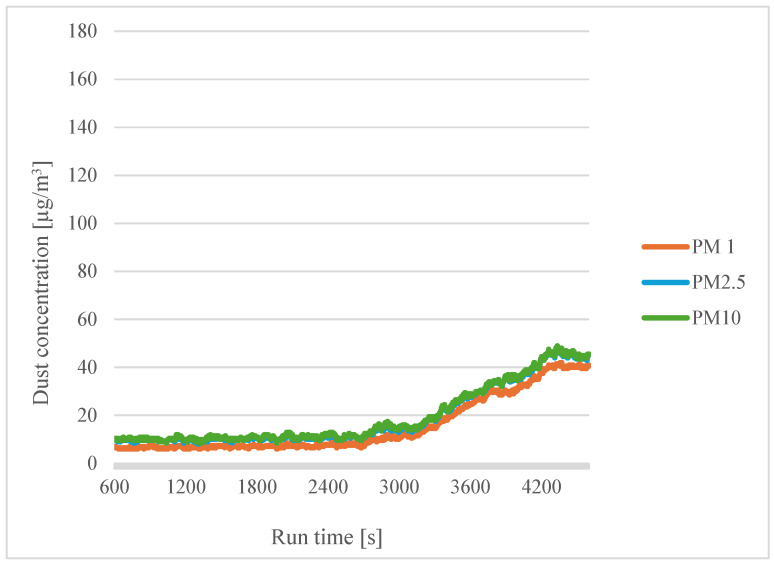
Dependence of dust concentration increase on process duration for different particle sizes for a printing temperature of 200 °C with dry filaments.

The following graphs show the difference in particle size for different filament moisture contents. Comparisons were made for a printing temperature of 200 °C and a dry filament (Figure 10).

The concentrations of the different particle sizes were similar to each other, but the concentration of PM1 was the smallest, which may mean that the smaller particles agglomerate into larger ones. This conclusion was reached by Zhou et al. [29].

**Figure 11:**
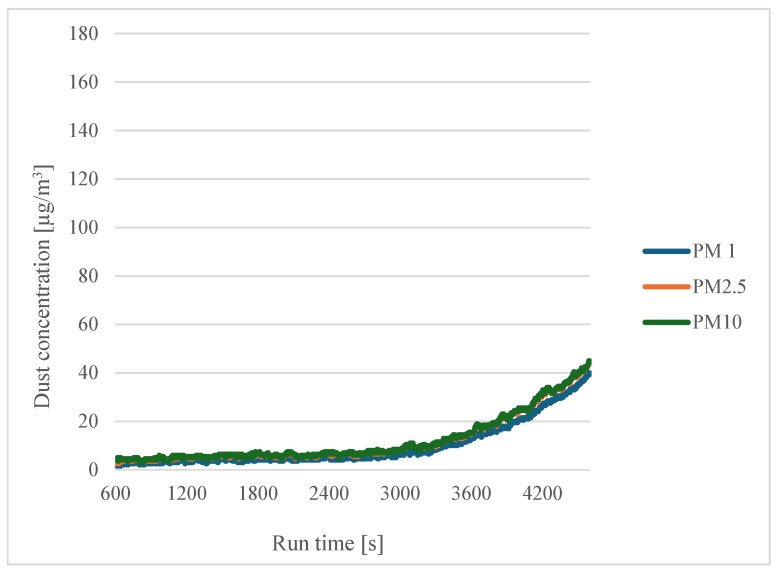
Dependence of dust concentration increase on process duration for different particle sizes for a printing temperature of 200 °C with filaments soaked in distilled water for three hours.

**Figure 12:**
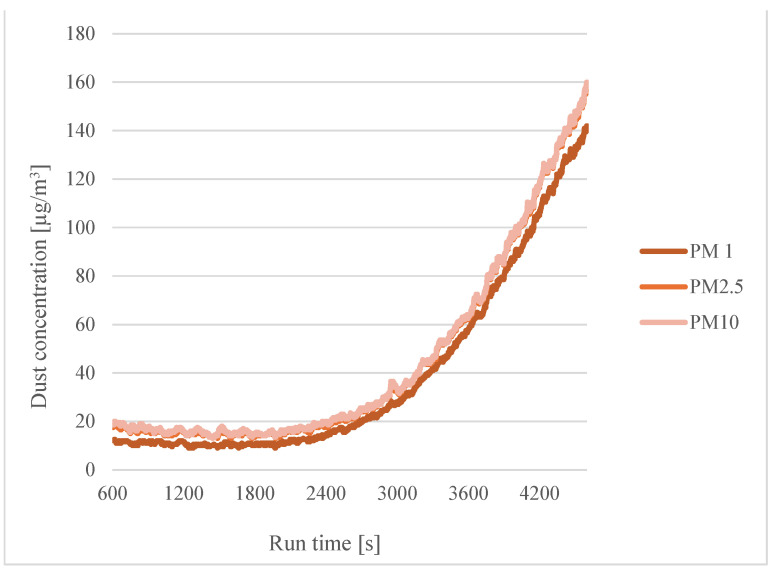
Dependence of dust concentration increase on process duration for different particle sizes for a printing temperature of 200 °C with filaments soaked in distilled water for twelve hours.

For the wet filaments (Figure 11 and Figure 12), a similar trend to the dry filaments was also observed.

For the other printing temperatures, similar comparisons were made, and the PM1 particle concentrations were also lowest regardless of the moisture content of the filament.

Based on the data obtained from the experiment investigating the effect of filament humidity on dust particle concentration, the following conclusions can be drawn:

An increase in filament humidity leads to higher dust emissions. The average dust emission values were lower for filaments with 0.18% humidity, ranging from 59 to 270 µg/m^3^ (sensor 1) and 77 to 378 µg/m^3^ (sensor 2). At a higher humidity level of 0.61%, emissions increased, averaging from 51 to 95 µg/m^3^ (sensor 1) and 79 to 122 µg/m^3^ (sensor 2). The highest emission values were recorded at 0.83% humidity, with the average levels reaching 284 µg/m^3^ (sensor 1) and 325 µg/m^3^ (sensor 2).

The variability in results also increased with humidity. High standard deviations at 0.83% humidity (up to 173 for sensor 1 and 198 for sensor 2) indicate instability in the dust emissions at this humidity level, likely due to complex interactions between the moist filament and the printing process.

Anomalies were observed at 0.61% humidity. In one trial at this humidity level (experiment 5, repetition 3), particularly high values were recorded (705 µg/m^3^ for sensor 1 and 905 µg/m^3^ for sensor 2), suggesting possible influence from additional factors or instability in the dust emissions at this specific humidity.

In summary, the results indicate that filament humidity significantly affects dust emissions, with higher humidity levels leading to increased emission rates and greater variability.

## 4. Conclusions

The tests carried out made it possible to assess the influence of the moisture content of the filament on dust emission in the incremental manufacturing process. Based on this, it was possible to formulate conclusions and record observations about the experiment performed. It was observed that the amount of dust emission during incremental manufacturing is influenced by humidity and ambient temperature. An increase in printing temperature results in an increase in particle emissions, so for PLA printing, a maximum temperature of 200 °C is recommended. In the case of printing temperatures of 185 °C and 200 °C, no significant differences were observed for particle emission levels, so on this basis, the moisture content of the filament does not contribute to either improving or worsening particle concentrations. In the case of 215 °C, the filament with a higher moisture content has a positive effect on dust emission, i.e., it causes lower dust emission. Due to the higher standard deviation values for the wet filament, it can be seen that the material absorbs water to different extents. The greatest difficulty in the experiments carried out was the need to maintain the correct duration of soaking the filament in distilled water. The repeatability of the steps during the experiment made it easier.

Additional analysis of the results suggests that increased filament humidity generally leads to higher dust emissions and greater variability in measurements. When a moist filament is heated, the water it contains evaporates, creating micro-bubbles within the material. These bubbles expand rapidly under heat, causing disruptions and micro-ruptures in the filament structure, which release fine particles into the air and increase dust emissions. This degradation process contributes to the higher particle concentration observed with humid filaments.

Furthermore, the presence of water in the filament can cause inconsistencies in the extrusion process. Moist filaments may have variable viscosity, causing fluctuations in the material flow through the nozzle, which leads to irregular dust emissions and contributes to increased standard deviations in the results. In addition, water absorbed by the filament can accelerate hydrolysis, a chemical degradation process in which the polymer chains of PLA break down into smaller fragments due to moisture and high temperature. These smaller fragments become additional sources of fine dust particles.

The inconsistency in emissions can also stem from non-uniform water absorption within the filament, which may result in areas with a higher moisture content that emit more particles during heating. This uneven moisture distribution within the filament creates localised emission spikes, increasing variability in the dust measurements. In summary, higher filament humidity influences the physical and chemical stability of the material during printing, resulting in higher, less predictable dust emissions that vary according to moisture content and the filament’s thermal response.

## Figures and Tables

**Table 1:** Amount of formaldehyde, PM10 and PM2.5 released during printing at 200 °C [26].

3DP Workspace [µg × min/m^3^]			First Attempt			Second Attempt		
			Lack of Ventilation	Ventilation	Difference	Lack of Ventilation	Ventilation	Difference
FFF	PLA	Formaldehyde	1447.73	892.52	555.21	1639.63	1356.41	283.22
		PM10	987.43	988.84	−1.41	570.87	541.67	29.19
		PM2.5	527.31	527.88	−058	307.42	293.23	14.18

**Table 2:** Properties of Spectrum Premium PLA filament [37].

Parameter	Value
Material	Premium PLA
Colour	Deep black
Weight	1 kg
Diameter	1.75 mm
Ovality	0.53%
Printing temperature	185–215 °C
Date of production	21 January 2023

**Table 3:** Soaking time and moisture content of the filaments.

Sample No.	Soaking Time [h]	Moisture Content [%]
1, 2, 3	0	0.18
4, 5, 6	3	0.61
7, 8, 9	12	0.83

**Table 4:** Properties of the Anycubic S printer [39].

Parameter	Value
Printing technology	FDM
Minimum layer height	50 µm
Print area	210 × 210 × 205 mm
Filament diameter	1.75 mm
Maximum printing temperature	260 °C
Number of nozzles	1
Nozzle diameter	0.4 mm
Maximum nozzle speed	100 mm/s
Maximum platform temperature	110 °C
Materials used for printing	PLA, ABS, PTU, HIPS
Supported file types	STL, OBJ, JPG, PNG

**Table 5:** Determination of test samples.

Experiment Number	Repetition	Print Temperature [°C]	Filament Soaking Time [h]	Moisture Content of the Filament [%]
1	1	185	0	0.18
	2	185	0	0.18
	3	185	0	0.18
2	1	200	0	0.18
	2	200	0	0.18
	3	200	0	0.18
3	1	215	0	0.18
	2	215	0	0.18
	3	215	0	0.18
4	1	185	3	0.61
	2	185	3	0.61
	3	185	3	0.61
5	1	200	3	0.61
	2	200	3	0.61
	3	200	3	0.61
6	1	215	3	0.61
	2	215	3	0.61
	3	215	3	0.61
7	1	185	12	0.83
	2	185	12	0.83
	3	185	12	0.83
8	1	200	12	0.83
	2	200	12	0.83
	3	200	12	0.83
9	1	215	12	0.83
	2	215	12	0.83
	3	215	12	0.83

**Table 6:** Properties of the Plantower PMS3003 sensor [46].

Parameter	Value
Power consumption	Up to 100 mA
Range of measurements	
PM1	To 1.0 µm
PM2.5	To 2.5 µm
PM10	To 10 µm
Sensitivity	50% for 0.3 µm
	98% for 0.5 µm and above
Operating temperature	From −5 °C to 60 °C
Response time	Under 10 s

**Table 7:** Test results.

Experiment Number	Repetition	Difference 4600-0		Difference 4600-0		
		Sensor 1	Sensor 2	PM1.0 [µg/m^3^]	PM2.5 [µg/m^3^]	PM10 [µg/m^3^]
1	1	16	23	5	7	8
	2	138	172	47	54	50
	3	23	36	10	10	5
Average 1		59	77	20	24	21
Standard deviation		69	83	23	26	25
2	1	56	80	23	23	23
	2	87	134	36	37	38
	3	97	144	38	41	42
Average 2		80	119	32	34	34
Standard deviation		21	34	8	9	10
3	1	285	383	106	114	114
	2	241	356	96	102	102
	3	285	396	109	116	116
Average 3		270	378	104	110	111
Standard deviation		25	20	7	8	8
4	1	117	141	40	45	45
	2	47	63	18	19	19
	3	120	163	46	48	48
Average 4		95	122	35	37	37
Standard deviation		41	53	15	16	16
5	1	14	27	7	8	6
	2	100	131	37	40	39
	3	705	905	256	275	275
Average 5		57	79	22	24	23
Standard deviation		61	74	21	23	23
6	1	86	153	40	41	40
	2	40	64	16	17	19
	3	27	48	12	13	13
Average 6		51	88	22	24	24
Standard deviation		31	57	15	15	14
7	1	54	60	17	20	20
	2	12	21	6	6	6
	3	105	122	36	39	39
Average 7		57	68	19	22	22
Standard deviation		47	51	15	17	17
8	1	155	211	57	64	63
	2	312	287	23	128	149
	3	386	478	135	149	148
Average 8		284	325	72	113	120
Standard deviation		173	198	56	68	73
9	1	79	99	25	32	32
	2	84	131	36	36	36
	3	61	90	24	26	27
Average 9		75	107	28	31	32
Standard deviation		12	22	7	5	5

## Data Availability

The data presented in this study are available on request from the corresponding author. The data are not publicly available due to privacy restrictions.

## Abbreviations

3D	Three-Dimensional
FDM	Fused Deposition Modelling
FFF	Fused Filament Fabrication
PM1	Particulate Matter 1 Micrometre Or Less In Diameter
PM2.5	Particulate Matter 2.5 Micrometres Or Less In Diameter
PM10	Particulate Matter 10 Micrometres Or Less In Diameter
VOC	Very Volatile Organic Compound
VVOC	Volatile Organic Compound
SVOC	Semi-Volatile Organic Compound
ABS	Acrylonitrile Butadiene Styrene
HIPS	High-Impact Polystyrene
PTU	Thermoplastic Polyurethane Elastomer
PLA	Polylactic Acid
PVA	Polyvinyl Alcohol
PA	Polyamide
TGA	Thermogravimetric Analysis
EGA	Evolved Gas Analysis
VOC	Volatile Organic Compound
MMA	Methyl Methacrylate
NIOSH	National Institute For Occupational Safety And Health
STL	File Format: Standard Triangle Language
OBJ	File Format: Geometry Definition
JPG	File Format: Joint Photographic Experts Group
PNG	File Format: Portable Network Graphic
PMS	Smart Particulate Matter Sensor
DHT	Digital Temperature And Humidity Sensor
